# Melatonin prevents age-related hearing loss in the murin experimental model

**DOI:** 10.1016/j.bjorl.2022.06.002

**Published:** 2022-06-14

**Authors:** Lucieny Silva Martins Serra, Juliana Gusmão de Araújo, Gabriela Novanta, Lucas Lauand, Eduardo Magalhães da Silva, Selma Aparecida Souza Kückelhaus, André Luiz Lopes Sampaio

**Affiliations:** a Universidade de Brasília, Faculdade de Medicina, Laboratório de Pesquisa em Otorrinolaringologia, Brasília, DF, Brazil; b Universidade de Brasília, Faculdade de Ceilândia, Departamento de Fonoaudiologia, Brasília, DF, Brazil; c Universidade de Brasília, Faculdade de Medicina, Departamento de Morfologia, Brasília, DF, Brazil

**Keywords:** Melatonin, Age related hearing loss, Outer hair cells

## Abstract

**Objective:**

The present study aimed to perform a morphological and morphometric analysis of cochlear structures of C57BL/6J mice receiving oral melatonin for a 12-month period.

**Methods:**

32 male C57BL/6J were divided into control and melatonin groups. Control received saline and ethanol solution and melatonin group, 50 μL of 10 mg of melatonin/kg/day orally for a 12-month period. After de experiment the animals were sacrificed into a 40% concentration of CO_2_ chamber, and the blades were morphological and morphometrically analyzed.

**Results:**

The melatonin group revealed a higher median density of viable cells (45 ± 10.28 cells/100 μm^2^, 31–73, vs. 32 ± 7.47 cells/100 μm^2^, 25–48). The median area of stria vascularis was 55.0 ± 12.27 cells/100 μm^2^ (38–80) in the control, and 59.0 ± 16.13 cells/100 μm^2^ (40–134) in the melatonin group. The morphometric analysis of the spiral ligament reveals a higher median of total viable neurons in the melatonin (41 ± 7.47 cells/100 μm^2^, 27–60) than in the control group (31 ± 5.68 cells/100 μm^2^, 21–44).

**Conclusion:**

Although melatonin is a potent antioxidant, it does not completely neutralize the occurrence of presbycusis; however, it may delay the onset of this condition.

**Level of evidence:**

3.

## Introduction

Age-Related Hearing Loss (ARHL), or presbycusis, is a highly prevalent condition arising because of the cumulative effects of aging on the auditory system. It is a bilateral, symmetrical, and slowly progressive sensory change that decreases the auditory threshold sensitivity and speech understanding abilities.^1^

Several studies correlated ARHL with cognitive decline. Whether hearing loss causes cognitive decline or people with cognitive decline are more susceptible to hearing loss, remains unclear. Nevertheless, in the older population, cognitive decline, early onset of dementia, social isolation, and depression are correlated with deafness.^2^, ^3^, ^4^

The mechanisms causing ARHL remain unclear; however, the apoptosis process due to Reactive Oxygen Species (ROS) accumulation seems to be the most common mechanism of cochlea cell death. In addition to the search for drugs to decrease or cure presbycusis, several studies have been developed to find other substances, supplements, or diets that may reduce the damage caused by ARHL. Herein, antioxidants are prominent due to their ability to slow the cell aging process.^5^, ^6^, ^7^

Melatonin (*N*-acetyl-5-methoxytryptamine) is the main hormone secreted in the pineal gland of mammals. It participates in several physiological functions, including sleep, circadian cycle, and regulation of immune and cardiovascular system. This substance was chemically characterized as an amphiphilic indolamine derived from tryptophan and could efficiently eliminate free radicals with remarkable antioxidant properties due to its ability to stimulate antioxidant enzymes in different tissues.^8^

The mechanism of action of melatonin reveals that it is an effective antioxidant. Moreover, melatonin acts as an electron donor in nonenzymatic processes, neutralizes most free radicals, and stimulates the activity of several antioxidant enzymes, such as superoxide dismutase, glutathione peroxidase, and glutathione reductase. Furthermore, it protects lipids, proteins, and DNA from oxidative damage, being highly concentrated in mitochondria.^8^, ^9^

Melatonin can prevent deafness and ototoxicity related processes, as reported in our previous study.^10^, ^11^ The present study aimed to perform a morphological and morphometric analysis of cochlear structures of C57BL/6J mice receiving melatonin for a 12-months period. This strain has a genetic mutation in the cadherin 23 gene that causes age-related deafness in an analogous way to humans.^12^

## Material and methods

### Study design

This experimental, prospective, and interventional study was conducted in 4-weeks-old 32 male C57BL/6J mice. The study was registered and approved by the Animal Research Ethics Committee, under protocol 57/2017.

### Study groups and treatment

Control group (CG, n = 16): animals treated with 50 µL of saline solution;

Melatonin group (MG, n = 16): animals treated with 10 mg/kg/day of melatonin (Sigma Aldrich®, St Louis, United States) previously diluted in ethanol, as described by the manufacturer.

The solutions were orally administered to all animals and were observed until they completely swallowed the solution.

### Experimental procedures

To evaluate the functional integrity of cochlea while developing presbycusis, the animals underwent Distortion Product Otoacoustic Emission (DPOAE) examination at 6, 8, 10, and 12 kHz. To obtain DPOAE data, the animals were anesthetized with ketamine hydrochloride (65 mg/kg) and xylazine (6.5 mg/kg), weighed, and conditioned in an acoustic booth. To obtain the distortion product (2f1–f2), two pure tones were used at a ratio of f2/f1 = 1.22, presented as the mean intensity of 65 dB SPL for f1 and 55 dB SPL for f2. The frequencies of 6, 8, 10, and 12 kHz were evaluated.

The animals received a daily oral dose of melatonin at a concentration of 10 mg/kg/day (Sigma Aldrich). At the end of 12-months of experiment, the animals were placed into a chamber with a concentration of 40% of CO_2_, resulting in their death by excessive Central Nervous System (CNS) depression and hypoxia due to oxygen displacement during alveolar gas exchange.

After euthanasia, the animals were perfused through the heart with a saline solution to remove the blood, and thereafter, 10% paraformaldehyde solution was injected to fix the tissues. Next, the heads were excised for histological studies. The anatomical specimens were processed according to the standard protocol of routine histological examination by immersing them in solutions with increasing alcohol concentrations for dehydration (70%, 80%, 90%, and 3 × 99.8%) for up to 1 h each, followed by three xylol baths for up to one hour each for fat removal (diaphanization), and were later added in Paraplast® synthetic resin (Sigma Aldrich).

Moreover, the specimens were blocked in two anatomical planes, transversal and sagittal. The microtomy was based on the surface of the tympanic bullae (anterior and lateral sides) and the histological sections were 6 μm thick in both sagittal and transversal planes; the slides were stained with Hematoxylin and Eosin.

The images were captured in mid-modiolar planes in an Aperio ScanScope® scanner under 20× magnification and were qualitatively analyzed by a single observer using the ImageScope software version 11.2.0.780 (Aperio Technologies Inc., Vista, CA, USA).

Furthermore, the slide photomicrographs were analyzed by a single blind observer to identify the groups. The structures such as hair cells, spiral ligament, stria vascularis, and spiral ganglion were assessed. The internal and external hair cells were qualitatively evaluated to verify the absence and the presence of these structures. The spiral ligament and spiral ganglion were analyzed based on the number of viable cells, and the stria vascularis was analyzed based on the area. Cell density analysis was performed by quantifying the viable cells present in each photomicrograph of the material, fractioned by the selected area.

### Analytical procedures

The normality of the variables was analyzed using the Kolmogorov-Smirnov test and the homogeneity of the variances using the Bartlett test. The *t*-test was used to compare the two groups with normal distribution, and the Mann-Whitney test was used for nonparametric data. Differences with *p* < 0.05 were considered as statistically significant. The analyses were performed using the Prism® 5 Software Package (GraphPad, USA, 2005).

## Results

**Figure 1:**
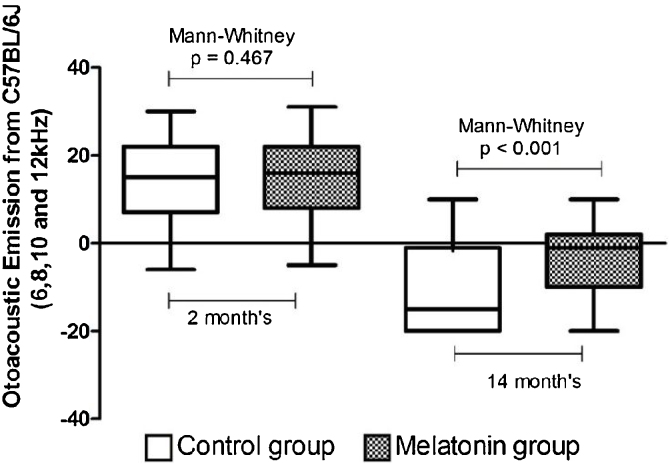
DPOAE amplitude values at 2 and 14 months of age in the frequencies of 6, 8, 10, and 12 kHz. White boxplot: control group. Hatched boxplot: melatonin group.

Fig. 1 illustrates the groups at 2 and 14 months of age. The two groups did not differ at the commencement of the experiment. At the end of the 12-month follow-up, the MG (10 mg/kg/day) presented higher DPOAE amplitude values.

### Structural assessment of the cochlea

It was not possible to perform a quantitative analysis of the external and internal hair cells due to the degeneration of these structures in both groups related to the age of the animal. The specimens showed good preservation in both groups.

### Spiral ganglion

The results revealed that the median density of viable cells in MG (45 ± 10.28 cells/100 µm^2^, 31–73) was higher than that in CG (32 ± 7.47 cells/100 µm^2^, 25–48) (Mann–Whitney, *p* < 0.001).

**Figure 2:**
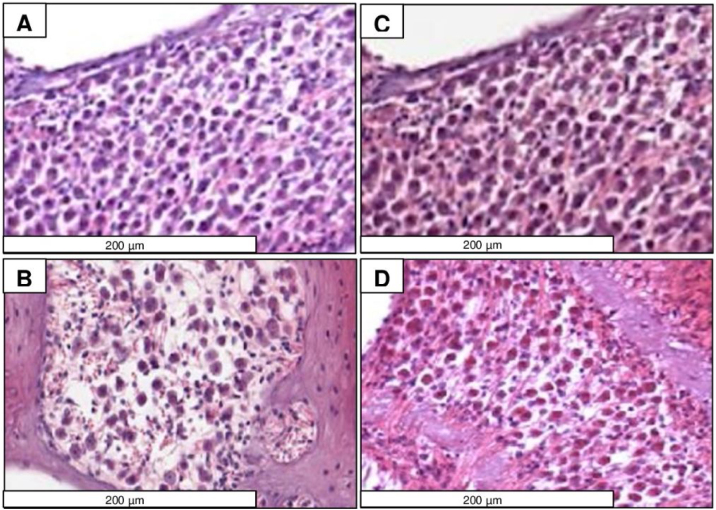
Photomicrographs of the spiral ganglion of CG (A and B) and MG (C and D) animals aged 2‒14 months. A and C were obtained from the apical region and B and D from the basal region of the cochlea. There is higher cell density in C and D compared to A and B. H&E staining.

Fig. 2 illustrates the photomicrograph of the spiral ganglion of a CG (A and B) and MG animal (C and D) treated for a period of 2–14 months. Photomicrographs A and C were obtained from the intermediate region and B and D from the basal region of the cochlea. There is a higher cell density in charts C and D (MG) compared to that in A and B (CG).

### Stria vascularis

**Figure 3:**
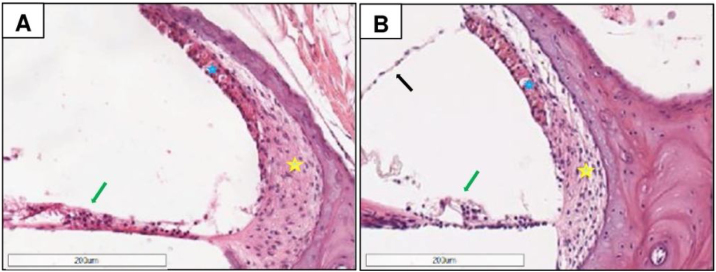
Photomicrographs of the cochlear duct of a CG (A) and an MG animal (between 2 and 14 months), revealing the stria vascularis (blue star), spiral ligament (yellow star), and organ of Corti organ (green arrow); the Reissner’s membrane is observed in B (black arrow). The morphometric results revealed that the cellular density in the stria vascularis does not differ between groups. H&E staining.

Fig. 3 illustrates the cochlear duct of a CG (A) and MG (B) animal. The morphometric results revealed that the viable cell density in the stria vascularis (blue star) does not differ between the two groups. The area was the criterion used in the morphometric analysis of stria vascularis, which revealed no difference between CG and MG. The median area of stria vascularis was 55.0 ± 12.27 cells/100 µm^2^ (38–80) in the CG, and 59.0 ± 16.13 cells/100 µm^2^ (40–134) in the MG (Mann–Whitney, *p* = 0.199).

The figure depicts the spiral ligament (yellow star) and the organ of Corti (green arrow). In B (MG), the organ of Corti can be more easily visualized with more preserved cells and the Reissner’s membrane also can be visualized (black arrow).

### Spiral ligament

**Figure 4:**
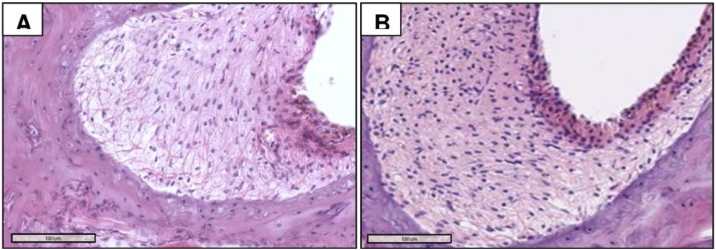
Photomicrographs of the spiral ligament of CG (A) and MG (2‒14 months) animals, with higher cell density in B. H&E staining.

The morphometric analysis of the spiral ligament (Fig. 4) reveals a higher median of the total viable neurons in the MG (41 ± 7.47 cells/100 µm^2^, 27–60) than in the CG (31 ± 5.68 cells/100 µm^2^, 21–44) (Mann–Whitney, *p* < 0.001).

## Discussion

In this study, the daily administration of 10 mg of melatonin/kg to animals susceptible to ARHL delayed the development of this condition, maintaining higher DPOAE values in the MG in all tested frequencies compared to CG. The morphometric analysis of cochlear structures revealed that the animals using melatonin had less cell degeneration compared to CG, expressed by the density of viable cells in the structures evaluated. No differences were observed between the groups for the variable stria vascularis area.

A previous study revealed the gradual hearing decline over time in both groups, which follows a similar pattern presented by humans in the ARHL process.^11^ The MG revealed better values during the cochlear function evaluation. Histopathological and morphometric evaluations were used to experimentally prove that this functional “protection” was associated with the preservation of cochlear tissues; however, the use of melatonin throughout the life of these animals did not prevent the onset of the ARHL process.

ARHL is a multifactorial (environmental and genetic) change, which presents with bilateral hearing loss caused by progressive degeneration of cochlear structures, such as sensory cells, stria vascularis, spiral ganglion, spiral ligament, as well as degeneration of central auditory pathways.^13^

It was not possible to perform a quantitative analysis of the internal and external hair cells, nevertheless, in some samples, it was possible to capture the images of these structures, as illustrated in Fig. 3.

A study on the identification of cochlear structures in C57BL/6J revealed that this animal already presented clear signs of hair cell degeneration at 50 days of life.^14^

The hearing loss seen in this lineage of mice is associated with degeneration of hair cells, which is a characteristic of sensory presbycusis.^15^ This includes progressive loss of age-dependent inner (IHC) and Outer Hair Cells (OHC) from the base to the apex of the cochlea, with OHC loss at the base of the cochlea starting from 3-months of age.^12^ One hypothesis for the initial onset of cell loss at the base of the cochlea may state that this gyrus is the first place where the sound comes into contact with the cochlea more intensively; thus, these cells would be more exposed to the damage caused by noise exposure, in addition to the oxidative stress that normally occurs during the cell aging process.

A study conducted to analyze macrophages in cochlear cells of C57BL/6J mice reported that age-related sensory cell degeneration started at the base of the cochlea with apical progression. In young mice (1-month), little or no degeneration was observed. Those with intermediate age (3–5 months) presented cell loss initially at the base of the cochlea, further progressing to the intermediate regions. In older animals (12-months), the sensory lesions expanded with complete OHC loss at the base region and significantly increased apex loss. According to the same study, these findings are in accordance with previous studies.^16^ In this study, it was not possible to perform a temporal analysis for the loss of these cochlear sensory cellular elements due to limited cells found while euthanizing the animals at 14-months of age; however, it was possible to identify cells in the upper gyrus of the cochlea in some blades analyzed. New studies based on electron microscopy analysis may contribute to the evaluation of these remaining cells to better understand the process of cochlear hair cell loss with aging.

The loss of hair cells in the animal model used occurs due to a mutation in the cadherin 23 gene.^12^ Cadherins are a class of transmembrane proteins that depend on calcium for their adhesion. In the inner ear, otocadherin is responsible for encoding a glycoprotein in the sensory epithelium that is necessary for the formation and function of stereocilia bundles. Due to this genetic mutation, the stereocilia become fragile, thereby causing early hair cell loss. The use of melatonin positively interferes with the oxidative destruction of proteins,^17^ as this substance is part of the ubiquitin proteasome system regulators that control protein degradation. Thus, the hair cells found in the MG reveal the protective effect of this substance on the cell aging process.

The viable cell density in the spiral ganglion of the animals at 14-months of age was effectively reduced in CG compared to MG; however, in both groups, the spiral ganglion was considerably degenerated. The spiral ganglion acts as the relay station of auditory information between hair cells and the CNS, and the loss of these neurons is common in neural presbycusis cases.^15^, ^18^ Other studies reported that in this lineage of mice, cell loss was observed in the spiral ganglion at 7-months of age.^12^

The death mechanisms of spiral ganglion neurons were partially characterized. A previous study supported the hypothesis that the loss of these age-related neurons involves intrinsic and extrinsic apoptotic pathways and may or may not be associated with the loss of hair cells. Aging inhibits Bcl-2 and activates Bax genes, which are key factors in the intrinsic pathway of apoptosis, and these further trigger extrinsic pathways through caspase activation.^18^ Thus, melatonin, as a mitochondrial antioxidant, penetrates cells delaying the apoptosis process, and consequently, the loss of spiral ganglion neurons, as seen in the present study. Another hypothesis is based on the fact that the loss of cochlear hair sensory cells due to lack of afferent stimuli may lead to atrophy and retrograde degeneration of spiral ganglion cells.

In addition to decreased viable cells in the spiral ganglion, age-related changes are observed in the spiral ligament and stria vascularis.^12^

Considering the spiral ligament, the density of viable cells was statistically higher in the MG than in CG at 14-months of age. A study performed on C57BL/6J cochleae revealed that this lineage presents cell loss in the spiral ligament associated with type IV fibrocytes. In 7-month-old animals, the authors reported significant loss of spiral ligament fibrocytes throughout the apical half of the cochlea, without significant hair cell loss. The same study demonstrated that cell loss in the spiral ligament precedes OHC and IHC loss.^19^ The present study corroborates these findings, since at 14-months, the spiral ligament, despite indicating clear degeneration in both groups, has virtually no hair cells. In the present study, the use of melatonin delayed cell loss in the spiral ligament compared to the CG.

In the present study, no differences were observed between groups while analyzing stria vascularis area. One hypothesis for this finding is that at 14-months of age enough degeneration reducing the area of stria vascularis was not observed under light microscopy. Several studies report the occurrence of stria vascularis thinning in these animals with aging.^1^, ^19^ This thinning presumably occurred in the samples analyzed in this study; however, the use of melatonin does not influence the stria vascularis area. The viability of the cells in this tissue was not analyzed; hence, future studies should better evaluate this structure with electronic microscopy techniques.

Melatonin is presently used as a natural supplement for regulating sleep in humans, with daily oral administration. Some studies revealed that, in addition to its circadian regulation function, this substance is an efficient free radical eliminator, with an important role in protecting cells from aging and from some neurodegenerative diseases.^20^ It is an antioxidant concentrated in the mitochondria, an organelle in which free radicals are produced in abundance; thus, it has an advantage due to its cellular location compared to other antioxidants. Besides its privileged location, melatonin also possesses metabolites that function as ROS kidnappers. Thus, while other antioxidants eliminate a single metabolite with a ROS clearance effect through a reaction cascade, melatonin and its metabolites can eliminate more ROS.^21^ Thus, melatonin presents effective systemic action in this experiment, thus proving to be a promising alternative in reducing the auditory system cellular damage caused by aging; however, complete elimination of cellular damage by aging in this animal model was not observed, and further studies are required to explore other such compounds.

Several strategies have been used to combat or prevent the action of aging on hearing. Aldosterone is a candidate drug for ARHL treatment due to the action of mineralocorticoids that can inhibit cell apoptosis, and calorie restriction also seems to be a good path for being directly related to insulin pathway modulation in the body aging process.^22^

The melatonin dose and form of administration used in this study should be highlighted. The oral dose of 10 mg/kg/day was administered, which was in line with the clinical dose used by humans. Several studies revealed antioxidant administration through food, water, or via subcutaneous implantation.^6^, ^23^, ^24^ Administering the antioxidant directly in the animal’s mouth, followed by swallowing ensured the ingestion of the exact volume offered daily throughout 14-months, and the animal returned to its box after ingesting the complete volume administered.

Considering numerous users of this substance for circadian cycle regulation, an observational analytical study should be conducted on these effects on the hearing system of habitual melatonin users compared to the nonusers with auditory evaluation using the DPOAE methodology.

The most important limiting factor in this study was lack of serial morphometric and histological analyses during the commencement of the study and over time to better understand the effects of aging on the hearing system and the possible beneficial actions of melatonin. Future studies should involve cellular ultrastructural analysis with electronic microscopy for a more detailed documentation of the aging changes in the cochlea of this species and should propose other protective agents with better effects than those proposed for melatonin.

## Conclusion

Although melatonin is a potent antioxidant, it does not completely neutralize the occurrence of presbycusis; however, it may delay the onset of this condition, as demonstrated in this study. It is necessary to develop more studies seeking for alternatives to treat ARHL, since this condition directly affects the quality of life of older people.

## Author’s contribution

LSMS participated in the study design, data collection, analysis, discussion, writing and critical review of the manuscript; JGA participated in the study design, analysis, discussion, writing and critical review of the manuscript; GN participated in the discussion, writing and critical review of the manuscript; LL participated in the study design, analysis, discussion and, writing critical review of the manuscript; EMS participated in discussion and critical review of the manuscript; SASK and ALLS participated in the study design, analysis, discussion, writing and critical review of the manuscript.

## Conflicts of interest

The authors declare no conflicts of interest.
